# The role of immunity in insulin resistance in patients with polycystic ovary syndrome

**DOI:** 10.3389/fendo.2024.1464561

**Published:** 2025-01-22

**Authors:** Qixuan Zhang, Zhe Yang, Xiangyang Ou, Mengying Zhang, Xiangyu Qin, Gengxiang Wu

**Affiliations:** ^1^ Reproductive Medicine Center, Renmin Hospital of Wuhan University, Wuhan, China; ^2^ Department of Oncology, Renmin Hospital of Wuhan University, Wuhan, China

**Keywords:** polycystic ovary syndrome, insulin resistance, immune molecules, immune cells, endocrine disorder

## Abstract

Polycystic ovary syndrome (PCOS) is a prevalent disorder of the endocrine system with significant clinical implications, often leading to health complications related to adipose tissue accumulation, including obesity, insulin resistance (IR), metabolic syndrome, and type 2 diabetes mellitus. While the precise pathogenesis of PCOS remains unclear, it is now recognized that genetic, endocrine, and metabolic dysregulations all contribute significantly to its onset. The immunopathogenesis of PCOS has not been extensively explored, but there is growing speculation that immune system abnormalities may play a pivotal role. This chronic inflammatory state is exacerbated by factors such as obesity and hyperinsulinemia. Therefore, this review aims to elucidate the interplay between IR in PCOS patients, the controlled immune response orchestrated by immune cells and immunomodulatory molecules, and their interactions with adipocytes, hyperandrogenemia, chronic inflammation, and metabolic homeostasis.

## 1 Introduction

**Figure 1:**
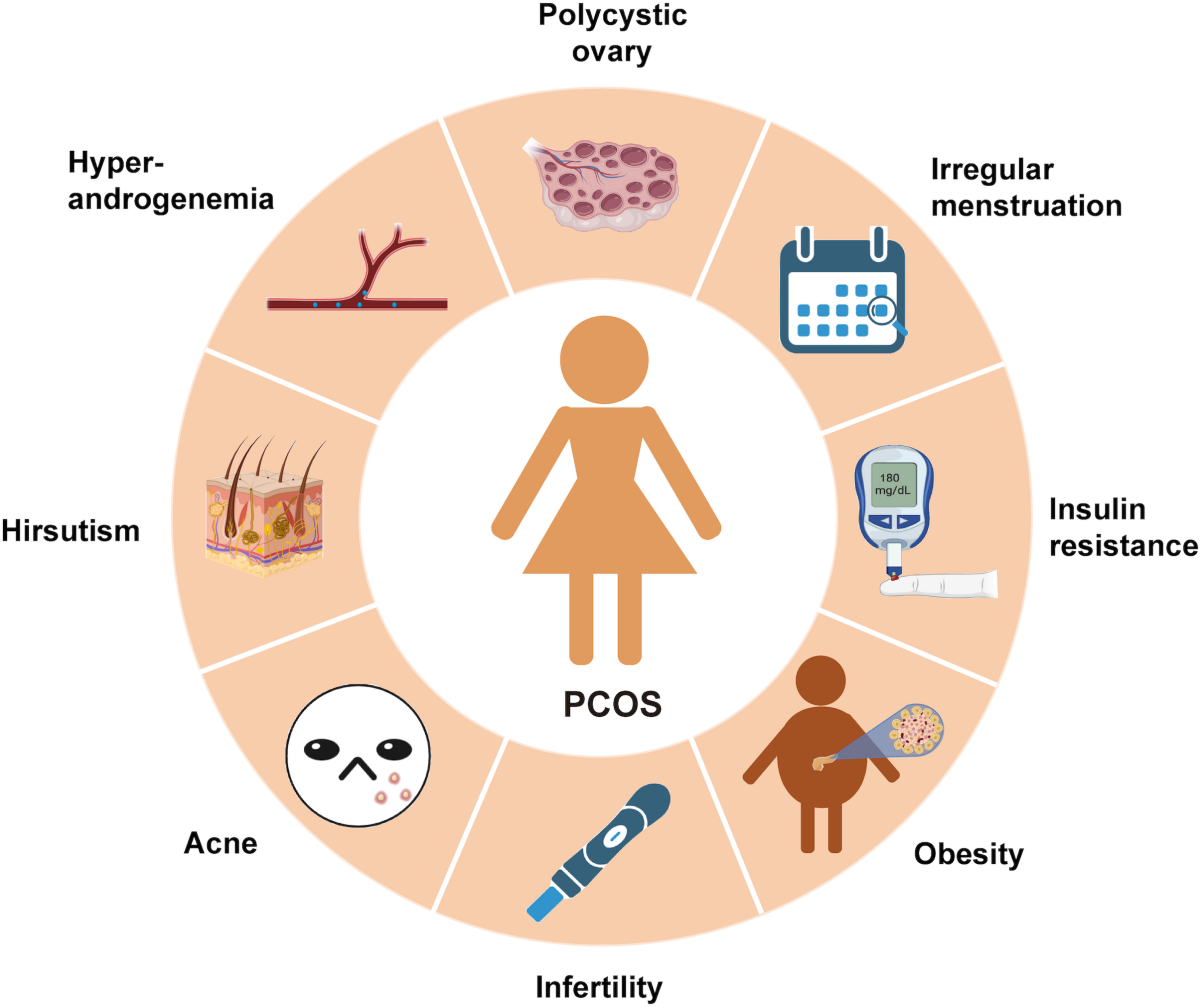
The symptoms of PCOS: PCOS is a chronic endocrine, metabolic and reproductive disorder with multiple signs and symptoms. Its main characteristics are ovulation dysfunction (manifested by irregular menstruation, such as oligomenorrhea and amenorrhea), hyperandrogen (manifested by hypertrichosis and acne), polycystic ovary morphology, metabolic disorders (obesity and insulin resistance) and infertility.

Polycystic ovary syndrome (PCOS) is the most prevalent endocrine disorder in women, characterized by anovulatory subfertility. It affects approximately 5.6% of Chinese women of reproductive age and around 10% of women from other ethnic backgrounds (1, 2). Clinical features of PCOS include irregular menstruation, infertility, hyperandrogenemia, ovarian polycystic changes, and metabolic abnormalities such as obesity, insulin resistance (IR), and dyslipidemia (**Figure 1**). PCOS is recognized as a significant risk factor for T2DM (T2DM), cardiovascular disease, gestational diabetes, and gestational hypertension.

The etiology of PCOS remains elusive, and its pathogenesis is complex. According to the latest diagnostic criteria, PCOS can be diagnosed by meeting any two of the following criteria: clinical/biochemical hyperandrogenemia, ovulation disorders, and ultrasound evidence of polycystic ovarian manifestations/abnormal Anti-Mullerian Hormone (AMH) levels (1, 3). Heterogeneity in the diagnostic modalities of IR is evident, with studies demonstrating that fasting triglycerides can be used selectively in place of fasting glucose for the assessment of IR, and that the HOMA-IR index, glucose, and serum insulin levels do not provide a fully consistent measure of IR severity (4, 5). While IR is not a diagnostic criterion for PCOS, the oral glucose tolerance test (OGTT) has been utilized as a primary screening tool for PCOS patients. In recent years, two distinct subtypes of PCOS with varying biochemical profiles have been internationally defined (6). The reproductive subtype is characterized by elevated serum luteinizing hormone (LH) and sex hormone-binding globulin (SHBG) levels, typically with a normal body mass index (BMI) and insulin levels. On the other hand, the metabolic subtype is associated with high BMI and insulin levels, along with relatively low LH and SHBG levels.

**Figure 2:**
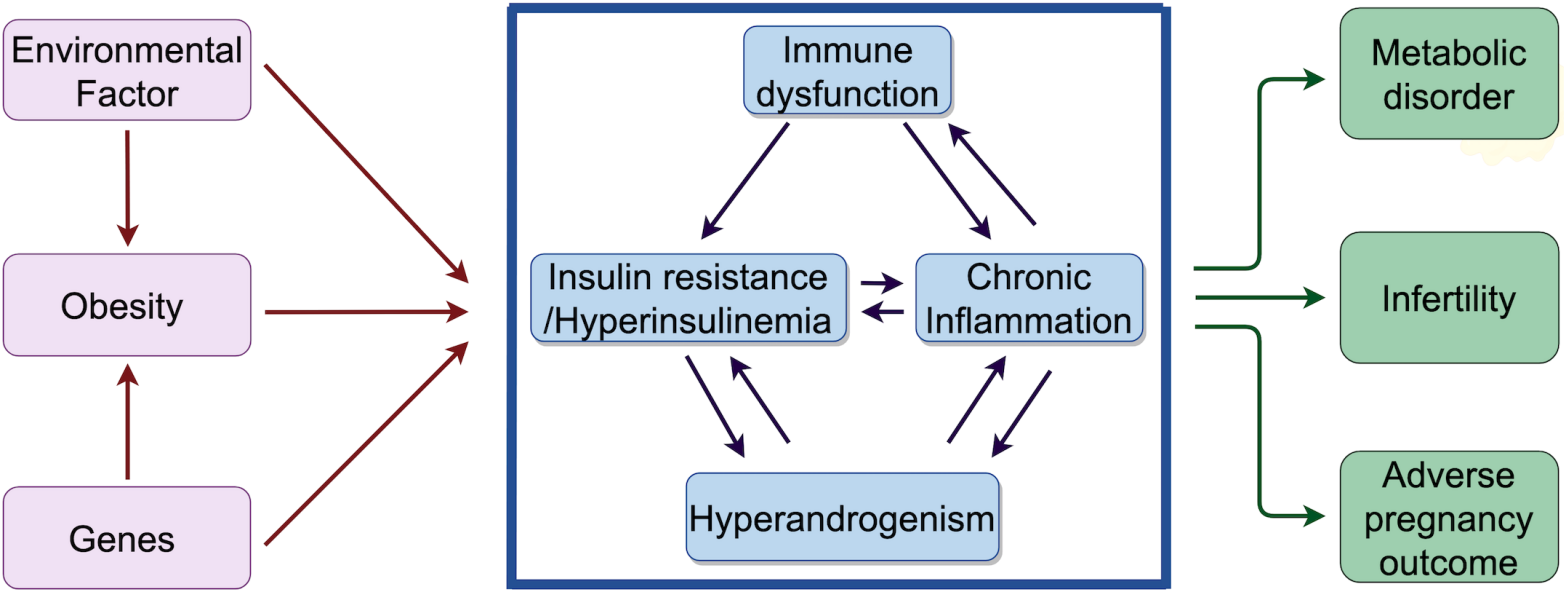
Factors affecting PCOS, phenotypes and mechanisms of interactions between long-term effects: mechanisms of interactions between IR, hyperandrogenemia and chronic inflammation in PCOS, and the relationship with long-term effects. Environmental and genetic factors may contribute to obesity, and the combination of obesity and these two factors predispose to IR and hyperandrogenemia.The interaction between IR and hyperandrogenemia may create a favorable environment for chronic inflammation, and the whole process is involved by the immune system. Ultimately, the interplay among chronic inflammation, immune system, IR, and hyperandrogenemia may result in infertility, metabolic disorders, and adverse pregnancy outcomes in patients with PCOS.

There is a growing body of evidence suggesting that immunity plays a significant role in the manifestation of symptoms in PCOS, particularly concerning insulin resistance, adipocyte dysfunction, glucose metabolism, and chronic inflammation. The chronic low-grade inflammation observed in PCOS patients is primarily linked to the accumulation of visceral adipose tissue, where adipocytes undergo necrosis due to hypoxia, leading to the infiltration of inflammatory cells that secrete various inflammatory cytokines. Immune cells have the capacity to either trigger or suppress inflammation by releasing pro-inflammatory or anti-inflammatory cytokines. Immune molecules such as antibodies complement proteins, and lymphokines are generated by immune cells in response to antigenic stimulation. Dysregulated immune cell function or imbalances in immune-related factors can result in immune dysfunction (7, 8). Due to impaired ovulation in patients with PCOS, IR causes androgen overproduction in patients with low progesterone levels, and the two inflammatory phenotypes reinforce each other. the IR-induced abnormalities persist and are exacerbated by androgenic and metabolic disorders, which induce severe immune dysregulation, resulting in the development of systemic symptoms in the patients (9) (**Figure 2**).

Moreover, several immune molecules are directly implicated in the immune response and are closely associated with insulin resistance in PCOS. This review provides a brief overview of current research on various innate immune cells and immune molecules, elucidating their roles and mechanisms in the development of insulin resistance in PCOS.

## 2 Immune cell regulation in IR

### 2.1 Macrophage immune regulation in IR

Macrophages play a crucial role in the immune response of patients with PCOS who develop IR. They serve as primary actors in innate immunity and also function as antigen-presenting cells in specific immunity. While not all patients with an IR phenotype show signs of obesity, it is evident that obesity significantly increases the risk of metabolic syndrome in individuals with PCOS (10). In obese PCOS patients with decreased levels of lipocalin (11), there is a substantial increase in both the size and quantity of adipocytes within a short timeframe. This rapid growth leads to local tissue ischemia and hypoxia, triggering the accumulation of macrophages, which can expand to more than five times their original size (12–14). Activated and differentiated M1 macrophages are predominantly located in a ring-like structure surrounding dead adipocytes, known as the crown-like structure (CLS), where they phagocytose these cells and sustain the release of pro-inflammatory cytokines (15).

Clinical studies have shown increased expression and phosphorylation levels of Forkhead box O1 (FOXO1) in peripheral blood macrophages from patients with PCOS (16). Insulin signaling in macrophages leads to the inactivation and nuclear exclusion of FOXO1 during its phosphorylation, a process that can be reversed under conditions of IR or inflammation (17). Silencing FOXO1 in macrophages results in monocytes exhibiting a greater polarization towards the M2-type, which helps to reduce the inflammatory state and IR (18, 19). On the other hand, upregulating FOXO1 promotes the release of Toll-like receptor 4 (TLR4)-mediated pro-inflammatory factors such as IL-1β, IL-6, and TNF-α (16).

TNF-α is recognized as the initial chronic inflammatory cytokine discovered in adipose tissue linked to obesity. TNF-α has the ability to diminish insulin expression by reducing the levels of insulin receptor substrate 1 (IRS-1) and glucose transporter 4 (GLUT4) proteins. Additionally, TNF-α can trigger inflammatory pathways like nuclear factor kappa-B (NF-κB) and c-Jun N-terminal kinase (JNK) in adipocytes. This activation hampers insulin sensitivity further under the influence of inflammatory factors, thereby worsening IR (20–22).

Obesity, inflammation, and IR show a mutually reinforcing relationship, i.e., increased accumulation of adipocytes promotes macrophage aggregation and differentiation, thereby inducing inflammation and stimulating IR.

In lean patients with PCOS, abnormalities in adipose tissue persist (11). In the subcutaneous adipose tissue of PCOS patients, there were higher levels of CD11c-positive adipose tissue macrophages, as well as elevated levels of TNF-α and leptin compared to body mass index (BMI)-matched women without PCOS. Additionally, in visceral adipose tissue, catecholamines exhibited significantly increased lipolytic effects, leading to the release of fatty acids from visceral adipose tissue, which were closely linked to insulin resistance. On the other hand, some studies have indicated that there is no significant difference in the transport and mechanism of action of fatty acids and monoacylglycerol in PCOS patients, regardless of whether adipocytes are normal or abnormal in size (23, 24).

Presently, the majority of studies concentrate on investigating the mechanism of IR in obese patients with PCOS. However, the cause of IR in lean PCOS patients might be associated with the pro-inflammatory differentiation of macrophages triggered by abnormal fatty acid lipolysis in visceral fat. It is noteworthy that the locations of macrophage aggregation in the two categories of PCOS patients are not identical (25). In patients with polycystic ovary syndrome (PCOS), there are elevated levels of androgens and serum homocysteine. In mice with PCOS induced by dehydroepiandrosterone (DHEA), homocysteine leads to an imbalance of M1/M2-type macrophages in adipose tissue, causing a shift from M2-type to M1-type macrophages. This transition exacerbates DHEA-induced IR. Furthermore, the abnormally elevated insulin levels at this stage can stimulate androgen secretion from ovarian membranous cells, reduce insulin receptor autophosphorylation in ovarian granulosa cells, and further worsen the chronic inflammatory response and IR in the ovaries (26, 27).

### 2.2 T Lymphocyte immune regulation in IR

T cells are a type of immune cell that matures in the thymus and plays a crucial role in specific immunity within an organism. They exhibit diverse subtypes and functions. CD4+ T cells contribute to maintaining the body’s immune response by recognizing major histocompatibility complex (MHC)-II-like molecules on the surface of antigen-presenting cells, subsequently activating and guiding other immune cells to the infection site. In the follicular fluid of patients with PCOS, the proportion of CD8+ T cells was notably lower compared to CD4+ T cells (28). CD4+ T cells play a more significant role in various clinical manifestations in PCOS patients, including IR. Based on their complex metabolic programming and cytokine production, CD4+ T cells can differentiate into distinct functional subpopulations. Among these, Th1 [producing interferon-gamma (IFN-γ)], Th17 (producing IL-17, IL-21, and IL-22), and Th2 (producing IL-4, IL-5, and IL-13) effector cells primarily rely on aerobic glycolysis for energy generation. In contrast, regulatory T cells (Treg) [producing IL-10, transforming growth factor β (TGF-β)] depend on oxidative phosphorylation driven by fatty acid oxidation (29–31).

In peripheral blood and adipose tissue of PCOS patients with metabolic abnormalities, T cells are the second largest immune cell population after macrophages, with significantly increased proportions of Th1, Th17, and CD8+ T cells and decreased proportions of Th2 and Treg cells (32–34). Metabolites of adipocytes can influence T-cell differentiation mediated by the T cell antigen receptor (TCR), and of the many soluble differentiation stimulators, fatty acids have been shown to be the strongest stimulators of Th1 differentiation (35, 36). T cells differentiated into pro-inflammatory Th1 cells produce IFN-γ, which induces macrophage differentiation towards the M1 phenotype. The aggregation of Th1 cells and infiltration of IFN-γ will recruit other immune cells, including macrophages, and increase the body’s inflammatory immune response (37). The above responses will be more pronounced in obese individuals. It was found that, in addition to its effects on immune cells, IFN-γ can directly induce IR in mature human adipocytes by taking charge of the activation of the janus kinase (JAK)/signal transducer and activator of transcription 1 (STAT1) pathway and inhibiting the expression of insulin signaling genes (GLUT4 and IRS1), lipid-forming genes (Perilipin, Lipoprotein Lipase, and Fatty Acid Synthesis Enzymes), and genes related to lipid storage (Recombinant Peroxisome Proliferator Activated Receptor(PPAG) and Lipocalin) (38). Insulin sensitivity was improved when the interferon gene was knocked out (39).

In the immunoregulation of T cells, the co-inhibitory receptors cytotoxic T-lymphocyte-associated protein 4(CTLA-4) and programmed cell death protein 1(PD-1) are co-expressed on effector T cells and participate in the dynamic balance of the immune response. The expression of PD-1 is maintained in T cells, which participates in the routine immune response of the body. In the acute phase, the expression of PD-1 decreases rapidly, and the body produces a rapid immune response. The proportion of T cells with elevated PD-1 expression in serum and follicular fluid was significantly higher in patients with PCOS than in normal patients, then metabolic disorders in patients with PCOS are associated with PD-1 expression (40). PD-1-overexpressing T cells are one of the subpopulations of the T-cell depletion phenotype (41, 42), and when in the presence of a PD-L1/PD-1 pathway blocking antibody, dendritic cells trigger the proliferation of syngeneic T cells and modulate T-cell subsets, which have elevated levels of IL-2 (43). This may suggest that in PCOS patients with abnormal T cells, PD-1 always plays a role in the relevant pathway and that high expression of PD-1 may inhibit T cell differentiation to Th2 type cells that secrete anti-inflammatory cytokines. In contrast, insulin has an anti-inflammatory effect on circulating immune cells, which may induce T cells to differentiate into an anti-inflammatory Th2 phenotype, and the dysregulation of the Th1/Th2 ratio and the PD-1-associated pathway may play a key role in resisting the effect of insulin on the immune response associated with T cells (44, 45).

CD4+ CD25+ Foxp3+ Treg cells were significantly lower in the peripheral blood of PCOS patients than in controls (34). FOXP3+ Treg cells can stimulate the production of IL-10 by macrophages through the secretion of IL-13, mediated by transforming growth factor-β. TGF-β and IL-10 act as the main anti-inflammatory cytokines in metabolism (46, 47). Smad4 and Recombinant Runt Related Transcription Factor 2 (RUNX2) genes are part of the TGF-β signaling pathway. The expression of these genes and related mRNAs increases during the development of hyperinsulinemia and IR in organisms (48). PCOS patients also differ from some obese patients in terms of hormone secretion levels. Estrogen helps limit inflammation, and in PCOS patients with hyperandrogenemia, the expression of the transcription factor B lymphocyte-induced maturation protein 1 (BLIMP1), which is androgen-dependent, is elevated. This leads to an immune response that brings about a new balance in the interaction between increased adipocyte inflammation and male-specific IL-33-producing stromal cells that actively recruit and locally expand Treg cell populations in a BLIMP1-dependent manner under the influence of sex hormones (49). In PCOS patients, there was a notable increase in Th17 cells, which predominantly secrete IL-17 to trigger neutrophilic inflammation, despite an overall decrease in Treg cells in the serum (34, 50). This indicates that the imbalance between Th17 and Treg cells in the context of abnormal insulin metabolism in PCOS patients is primarily due to the reduced levels of Treg cells.

### 2.3 B Lymphocyte immune regulation in IR

Similar to T-cells, B-cells are classified into different subpopulations based on their distinct phenotypes, functions, and the primary cytokines they secrete. Presently, there are two primary categories: B-1 and B-2 cells. B-1 cells are predominantly found in organ cavities, mucosal tissues, and adipose tissues. They can be further categorized into CD5+B-1a and CD5-B-1b based on their CD5 expression (51, 52). B-1a cells, as innate B-like cells, are the primary producers of natural IgM antibodies in the body (53), and mainly function in the absence of antigens. In contrast, B-1b cells participate in T-cell-mediated immune responses to external antigens. B-2 cells are B cells traditionally derived from the bone marrow and migrate to lymphoid organs (54). They generate and concentrate specific antibodies, playing a crucial role in humoral immunity, and have the ability to differentiate into memory B cells and plasma cells. When B cells secrete anti-inflammatory factors like IL-10, and IL-35, they are collectively known as regulatory B cells (Bregs). These cells are not distinct subtypes from B-1 and B-2 cells but rather functional subtypes within these two classes of B cells.

In the context of IR driven by inflammatory adipose tissue, B-cells are the initial immune cells to accumulate, followed closely by T-cells, and eventually by macrophages (55). In individuals with PCOS experiencing low-grade chronic inflammation, the levels of anti-inflammatory IL-10 produced by Bregs were significantly lower compared to controls (56–58). Conversely, enhancing IL-10 expression or administering it for short durations in IR mice led to a notable increase in systemic insulin sensitivity. This effect was lost in the absence of IL-10 (59–61), indicating its role in inhibiting the differentiation of T cells into pro-inflammatory Th1 cells.

In patients with PCOS, the levels of IL-10 produced by B-1 cells decrease, leading to an increase in T-cell differentiation towards a pro-inflammatory phenotype, consequently promoting the development of insulin resistance originating from visceral adiposity (62–64). Additionally, B-2 cells have been demonstrated to have a pro-inflammatory function in the expansion of adipose tissue in rats consuming a high-fat diet (65). Moreover, in PCOS patients, this activation of B cells is closely linked to hyperandrogenism and the androgen receptor (66).

In line with mouse data, hyperandrogenemia also increases levels of IL-1β, IL-8, and IL-18 in PCOS patients (67). IL-8 functions as a pro-inflammatory factor that attracts neutrophils to adipose tissue, contributing to inflammation and IR (68). Furthermore, in obese PCOS individuals, there are changes in the levels of the pro-inflammatory cytokine leptin. Leptin triggers the phosphorylation of JAK2, STAT3, p38MAPK, and ERK1/2 in B-cells, decreases the expression of apoptotic factors, enhances B-cell survival, and promotes Th1 cell differentiation (69, 70). Limited research has focused on the pro-inflammatory mechanisms of B cells in insulin-resistant PCOS patients. Additionally, the association between B cells and macrophage polarization mechanisms in dysfunctional adipose tissue requires further exploration.

### 2.4 Dendritic cells immune regulation in IR

Dendritic cells (DCs) are antigen-presenting cells that play a role in both innate and acquired immunity. They are categorized based on morphology into conventional DCs (CDCs) and plasma cell-like DCs (PDCs) (71). DCs express surface markers like MHCII, CD11b, CD11c, and C-X3-C motif chemokine receptor 1, which are shared with macrophages. This similarity indicates that in dysfunctional adipose tissue, DCs function akin to macrophages (72, 73). Unlike many other immune cells that undergo substantial proliferation, DCs proliferate within tissues, albeit to a lesser extent (74, 75).

CDCs in adipose tissue can be subdivided into cDC1 (CD4- CD8α+ CD103+ CD205+ CD11b- CLEC9A+ XCR1+ CD24+ MHCII-) (76–78) and cDC2 (CD4+/- CD8α- CD205-CD11b+ CLEC4A4+ CD24+ MHCII+) (79, 80) based on descent. Both types of DCs increase in obese or inflamed tissues. cDC1 cells lacking MHCII are involved in cross-presentation to CD8+ T cells, leading to an expansion of CD8+ cells (81). The lack of MHCII expression in cDC1 and the decrease in the total number of CD11c+ cells enhance the body’s insulin sensitivity and lower the likelihood of IR. Nevertheless, this evidence alone does not imply a direct involvement of CD11c+ DCs in IR; CD11c+ macrophages also contribute to the reduction in inflammation levels (77).

Additionally, human monocyte-derived dendritic cells (moDCs) express CD14+ and are considered precursors of inflammatory dendritic cells (82). Conventional dendritic cell type 2 (cDC2) triggers CD4+ T-cell differentiation and exhibits a protective effect against adipose tissue inflammation. This effect induces IL-10 production through the activation of the Wnt/CD11b-catenin pathway in cDC2 cells [CD11c(hi) MHCII+ CD11b-], which typically express MHCII. Conversely, the previous pro-inflammatory function can be suppressed in dendritic cells [CD11c(hi) MHCII+ CD11b+] by activating the PPARγ pathway (83). Both mechanisms may contribute to IR in patients with PCOS. Moreover, the influence of serum estradiol levels on gonadotropins in PCOS patients impacts the maturation of CD11c+HLADR+ dendritic cells in human follicular fluid, consequently affecting IR levels (84). However, the precise molecular mechanism underlying this process remains unknown.

### 2.5 Natural killer cell immune regulation in IR

NK cells, which are innate lymphoid-like cells (ILCs) originating from the bone marrow, are widely distributed in various organ tissues and serve specific immune functions. Studies have shown that NK cells constitute 13% of immune cells in visceral fat and contribute to the inflammatory polarization of the immune response (85, 86). Inflammatory conditions predominantly regulate the local activity of NK cells through IL-12, IL-15, and IL-18 produced by dendritic cells and macrophages, with a particular focus on the extensively researched role of IL-15 (87–89).

IL-15 is highly expressed in the follicular fluid of PCOS patients and is positively correlated with serum testosterone levels. Treatment with IL-15 enhances the expression of Cytochrome P450 17A1 (CYP17A1) in granulosa cells, a key enzyme for androgen synthesis. Elevated levels of CYP17A1 lead to increased production of DHEA, a precursor for most androgens. Elevated androgen levels significantly contribute to inflammation and inflammation-induced IR (90).

In an inflammatory state, the expression of adipocyte NKp46 (NCR1) ligand is upregulated, and IL-15 binds to IL-15Rα on NK cell membranes, activating NK cells to produce IFN-γ. This promotes the polarization of CD4+ cells and macrophages towards an inflammatory state, contributing to the development of IR. Aggregated macrophages secrete large amounts of chemokines such as C-C motif chemokine ligand 3(CCL3), C-C motif chemokine ligand 4(CCL4), and chemokine CXC ligand 10 (CXCL10), which facilitate the recruitment of NK cells (91, 92).

Adipose tissue macrophage inflammation and IR can be improved by inhibiting NK cell function using neutralizing antibodies or through E4bp4 heterozygous knockout in mice (93). This is because inhibiting NK cell function leads to decreased expression of TNF-α and IL-1β, while increasing the expression of anti-inflammatory IL-10 and Arg1. Furthermore, leptin and lipocalin, linked to obesity, also regulate NK cell function. Short-term exposure to leptin enhances NK cell cytotoxicity and IFN-γ levels, whereas long-term exposure has the opposite effect. Conversely, lipocalin consistently suppresses NK cell function, irrespective of treatment duration (94, 95). This data indicates that in obese patients with PCOS, elevated leptin levels and/or decreased lipocalin levels activate NK cells, promoting the initiation and progression of inflammatory responses in adipose tissue, partially elucidating the development of IR in obese PCOS patients.

Due to significant phenotypic diversity and individual variances in PCOS patients, limited research has focused solely on NK cells, their cytokines, and their precise mechanisms.

### 2.6 Granulocyte immune regulation in IR

Granulocytes are a type of leukocytes characterized by specific cytoplasmic granules. They are classified into major subgroups, including eosinophils, basophils, and neutrophils. In the complex inflammatory environment of PCOS patients, granulocytes are rapidly generated in the bloodstream, contributing to the immune response (96, 97). Specifically, the neutrophil-to-lymphocyte ratio (NLR) showed a positive correlation with HOMA-IR and serum insulin levels, irrespective of the obesity level in PCOS patients. This indicates a potential higher proliferation of granulocytes compared to lymphocytes in PCOS patients. Among the three types of granular leukocytes, neutrophil count and its proportion in leukocytes are commonly utilized as serum inflammation markers. Lourdes et al. observed that in PCOS patients with both hyperandrogenemia and hyperinsulinemia, the elevated white blood cell counts were primarily attributed to increased neutrophils. Moreover, glucose-lowering medications effectively suppressed the rise in neutrophils, thereby alleviating the inflammatory response in patients (98). Currently, it is believed that the mechanism of neutrophils affecting IR in PCOS is linked to myeloperoxidase (MPO). This belief stems from the higher MPO levels in leukocytes of PCOS patients compared to controls, with a more significant increase in the presence of IR (97, 99). However, due to the harsh culture conditions of granulocytes and the difficulty of detection, no authoritative study has yet revealed the specific mechanism of their role in IR in PCOS patients.

**Figure 3:**
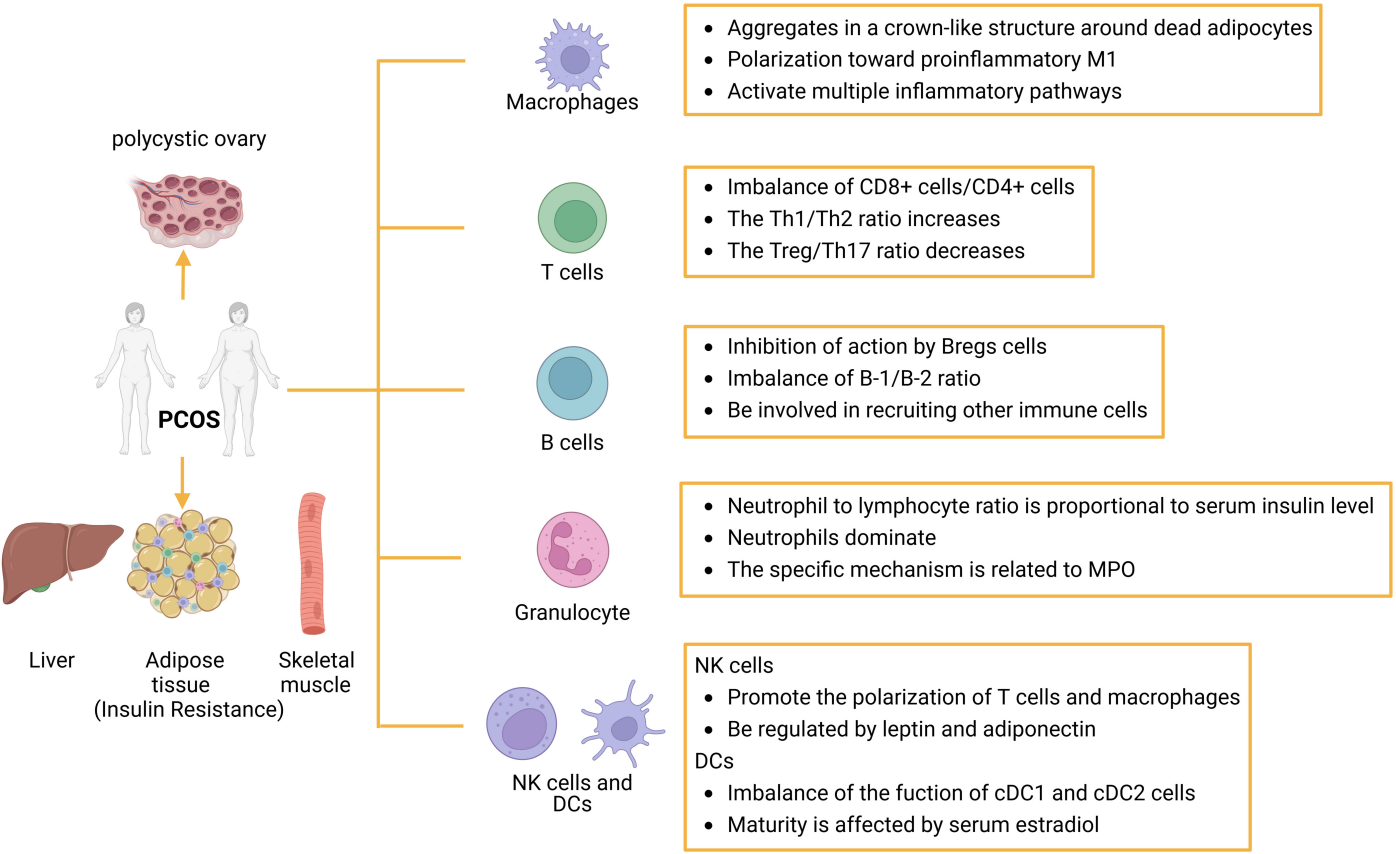
The role of immune cells in IR in patients with PCOS: Abnormal proliferation of immune cells can cause immune dysfunction or imbalance in the ratio of immune-related factors. Several immune cells are involved in the inflammatory response, such as macrophages, T cells, B cells, granulocytes, dc, and NK cells, which can initiate or inhibit the host’s inflammatory response through the production of proinflammatory cytokines or suppressor cytokines. dc, dendritic cell; NK, natural killer cell.

Overall, the mechanisms by which immune cells influence insulin resistance in PCOS patients are intricate and varied, necessitating further investigation into the functions of most immune cells (**Figure 3**).

## 3 Immune molecules regulation in IR

### 3.1 Cytokine

#### 3.1.1 Tumor necrosis factor-α in IR

The tumor necrosis factor (TNF) superfamily comprises 19 ligands and 29 receptors (100), all of which demonstrate proinflammatory activity. In the 1960s, a factor that induces tumor regression was discovered and named TNF-α. TNF-α was initially identified in macrophages and serves as a prototype of the TNF ligand family. Upon activation, TNF-α initially generates a transmembrane protein (tmTNF-α) in adipocytes and subsequently engages in signaling via two receptors, TNF-R1 and TNF-R2 (101, 102). Two transcription factors, NF-κB, and activator protein-1 (AP-1), have been identified to participate in the signaling process (103, 104). This factor was detectable in various components of the human oocyte-corona cumulus complex as early as fifty years ago (105, 106). Currently, TNF-α, a significant inflammation marker *in vivo* for PCOS patients, also impacts the clinical characterization of PCOS patients in diverse manners (107).

The molecular levels of GLUT-4 and IRS1, associated with glucose membrane transport and glucose uptake respectively, were significantly lower in PCOS patients with BMI compared to normal women (108–110). Conversely, TNF-α may contribute to insulin resistance in PCOS patients by inhibiting tyrosine kinase phosphorylation of IRS and reducing the biological activity of GLUT-4 (111). Statistically significant variations were also noted in the expression levels of TNF-α and lipocalin in obese women with PCOS compared to those without PCOS (112, 113). A negative correlation was observed between the two variables. *In vitro* experiments elucidate this relationship: TNF-α decreases lipocalin expression and secretion, while lipocalin influences TNF-α-induced proinflammatory and insulin inhibitory effects (114–117). It is evident that in patients with PCOS, TNF-α, known for its pro-inflammatory role, plays a predominant role. NF-κB, a transcription factor influenced by TNF-α, may also contribute to insulin resistance through a specific mechanism linked to its significant upregulation of inflammatory interleukins (e.g., IL-1β, IL-6) upon activation (118).

#### 3.1.2 Interleukin-6 in IR

Interleukin-6 (IL-6), a multifunctional signaling molecule, is primarily produced by immune cells, epithelial cells, and tumor cells (119, 120). In the field of reproductive endocrinology, its key roles include regulating gonadotropin secretion, implantation, luteal function, and embryo development. IL-6’s activity is triggered by various pro-inflammatory molecules like TNF-α, interferon-γ (IFN-γ), and IL-1 (121). Initial studies indicate that IL-6 may enhance lipolysis in human adipocytes cultured for 48 hours. Sennet et al. found that short-term IL-6 treatment (30-90 minutes) increased socs-3 expression in HepG2 cells and rat hepatocytes, potentially affecting insulin-induced IRS-1 tyrosine phosphorylation, p85 binding, and downstream PKB/Akt phosphorylation, with a similar mechanism implicated in T2DM (122–124). Higher serum IL-6 levels in normal-weight polycystic subjects suggest that elevated IL-6 levels in nonobese subjects with PCOS may also contribute to IR (125). Two other studies on purely obese patients and obese PCOS patients also found higher IL-6 expression in obese PCOS patients compared to purely obese patients (126, 127). Therefore, in obese conditions, androgens trigger immune responses in patients with polycystic ovaries, creating a mutually reinforcing relationship between polycystic changes, hyperandrogenism, and insulin resistance. The more complex mechanisms of IL-6 need further exploration.

#### 3.1.3 Interleukin-17 in IR

The pro-inflammatory cytokine IL-17 is associated with tissue inflammation induction. This cytokine family comprises six members, IL-17A to IL-17F, with IL-17A and IL-17F being closely related and co-expressed on linked genes. IL-17 is sourced from both Th17 and Th2 cells, while IL-17E is specifically from Th2 cells and enhances the Th2 pathway’s activity. Previous studies have demonstrated a significant increase in Th17 cells in PCOS patients, with high concentrations of IL-17A, a characteristic Th17 cytokine, compared to the control group.

IL-17 plays a role in adaptive immunity and in the production and interaction of innate immune cells on tissue cells, so it is reasonable to assume that it is involved in the low-grade chronic inflammation produced by adipose tissue as an important participating component. Furthermore, IL-6, a signaling molecule necessary for the differentiation of CD4+ cells into the Th17 lineage (128, 129), is a major downstream gene target of IL-17 (130, 131). A study by Fulghesu et al. showed that IL-6 concentrations were much higher in the sera of patients with the same PCOS who also had IR than in those without IR (132). Similar findings were obtained in obesity-promoting Th17 cells in expanded diet-induced obese mice, where Th17 cell numbers were significantly increased in wild-type genes, whereas the response of Th17 cells was not enhanced in IL-6-deficient mice (133). These studies suggest that, to some extent, the chronic inflammation that generates IR is dependent on the associated response of Th17 cells, which in turn is dependent on the activation of IL-6. CCAAT/enhancer binding protein (C/EBP) β and C/EBPδ are important transcription factors that promote adipocyte differentiation (134), and during adipogenesis, C/EPBβ is sequentially phosphorylated at three sites (Thr188, ser184 and Thr179) in its internal regulatory structural domain (135). Although IL-17 inhibits adipogenesis, it induces an increase in the abundance of C/EBPβ and C/EBPδ in lipogenic cell lines (130, 136) and induces phosphorylation at Thr188 and Thr179 sites (137). A study showed that IL -17 triggered a significant increase in IL-6 levels in differentiated adipocytes in culture of human mesenchymal cells (138). Increased IL-6 stimulates Th17 cell differentiation, which in turn may lead to an upregulation of IL-17-induced IL-6 levels. Although the relationship between IL-17 and IL-6 mutual stimulation has been demonstrated in many cells including adipocytes, the exact mechanism of IL-17 action on adipocytes and IR remains unknown, as does the promotion of CD4+ T cell differentiation into Th17 cells by IL-6.

#### 3.1.4 Interleukin-18 in IR

Interleukin 18 (IL-18), also known as IF-γ-inducible factor, is a pro-inflammatory cytokine that in humans is encoded by the IL-18 gene. IL-18 belongs to the IL-1 superfamily and is produced by macrophages and other cells. Upon IL-18 stimulation, natural killer (NK) cells and some T cells release another important cytokine, IFN-γ or type II interferon, which in turn activates macrophages or other cells.

In adipose tissue, non-adipocytes, such as stromal vasculature and immune cells, are the primary source of IL-18 (139, 140). Simultaneously, immune cells synthesize IL-18Ra/b heterodimeric receptor complexes, to which IL-18 binds and plays a crucial role. Abnormal inflammation in adipose tissue leads to changes in IL-18R/IL-18 expression on CLS immune cells (141). Studies on animals have shown that mice with reduced IL-18 secretion exhibit elevated levels of adipose and pro-inflammatory factors in adipose tissue, resulting in glucose intolerance (142). However, a statistical analysis of diabetic patients revealed a positive correlation between elevated serum IL-18 and glycemic abnormalities after excluding the influence of BMI and adipokines. Similar results were observed in patients with PCOS and other low-grade chronic inflammatory conditions (107, 143, 144). This variation may be attributed to the diverse functions of IL-18 in different tissues (145). For instance, in bone marrow tissues, IL-18 primarily contributes to maintaining glucose homeostasis, while in tissues other than bone marrow, it mainly regulates the expansion of adipocytes (142). In individuals with adipose inflammation, IL-18 expression showed a negative correlation with IRS1, GLUT-4, lipocalin, and PPARγ expression (142), while demonstrating consistent changes with the severity of obesity, insulin resistance, lipid metabolism, and dyslipidemia, all indicating a significant role of IL-18 in insulin metabolism abnormalities (146–148).

### 3.2 Immunomodulatory molecules in IR

#### 3.2.1 Interferon regulatory factor in IR

Up to now, studies have identified nine members of the mammalian interferon regulatory factor (IRF) family: IRF1, IRF2, IRF3, IRF4 (PIP, LSIRF, or ICSAT), IRF5, IRF6, IRF7, IRF8 (ICSBP), and IRF9 (ISGF3γ) (149, 150). IRFs play a key role in the regulation of innate and acquired immunity as a transcriptional regulator of type I interferons and interferon-inducible genes (151). The structure of IRF and its function in tumor growth have been well described (152). This study aims to clarify the potential relationship among IRFs, immune cells, and IR in patients with PCOS (as shown in **Table 1**).

**Table 1:** Summary of IRF members and their connection to IR in PCOS.

IRF	Expression	Target genes in immune cell	Functions	Connection to IR in PCOS
IRF1 (192–199)	Prevalently in multiple tissues and cell lines.	IFN-inducible genes (iNOS, Caspase-1, Cox-2 etc.) Connected with MyD88 and TLR signaling genes (IFN-β, IL-4, IL-12, IL-15, PPARy).	Acts to block the cell cycle and induce apoptosis; participates in NK cell development and CD8+ T cell differentiation; promotes T cell differentiation toward Th1; accelerates myocardial remodeling, Ischemia-Reperfusion(I/R)-induced liver injury and T1DM.	Enhances androgen sensitivity and worsens the body’s inflammatory response via the TGF-β/IRF1 signaling pathway; the pathway leading to IR in PCOS may resemble that in T2DM, with regulation by the troponin I type 3(TNNI3) and Baculoviral IAP Repeat Containing 3(BIRC3) genes.
IRF2 (200, 201)	Similar to IRF1	Attenuates type I IFN (Just like IRF1); Connected with MyD88 and TLR signaling genes(IL-4 and IL-12).	Influences type I IFN response; involved in NK cell development and CD4+ DC differentiation; promotes T cell differentiation toward Th1.	No studies have been done, the mechanism of action may be similar to that of IRF1
IRF3 (202–207)	Most tissues and cell lines.	Induces type I IFNs (IFN-α/β) DNA stimulation(ERK2, IKKβ, PPARγ) TLR stimulation Microglia stimulation(IL-22).	Induces type I IFNs; Maintains insulin sensitivity and lipid homeostasis; reduces inflammation and myocardial hypertrophy; participates in I/R injury tolerance induced by pretreatment with TLR ligands; exacerbates hepatic I/R injury.	IRF3 enhances adipose inflammation and IR through its downstream IKKβ/NF-κB and PPP2R1B-AMPK/AKT pathways. Activation of IFN-β/IL-10 induced the transformation of macrophages into M1 type in white adipose tissue. TLR3 and TLR4 upstream of IRF3 participate in proinflammatory lipogenesis and IR by activating IRF3. Upstream IL-22/IL-22R1 inhibits IRF3 from increasing M2-type macrophage differentiation and alleviating symptoms.
IRF4 (208–212)	Exists in cells with high energy metabolism and is abundant in lymphocytes.	Connected with IRF5, MyD88 and TLR signaling genes (IRF5, IL-4, CREB and SRF)	Retards IRF5 interaction with MyD88 and reduces tlr-dependent inflammation; required for differentiation of CD4+ DCs, pDCs and macrophages; involved in B-lymphocyte development.	Within the adipose tissue of obese PCOS patients may be associated with reduced macrophage secretion of pro-inflammatory factors (IL -1β and TNF -α).
IRF5 (213, 214)	Mainly in B-cells and DCs.	Connected with MyD88 and TLR signaling genes (TNF-α, IL-6, IL-12); Participates in metabolic disease(TGF-β1).	Participates in IR and obesity.	There are no detailed reports in the literature, but IRF5 levels are elevated in obese patients.
IRF7 (215–218)	Most tissues and cell lines.	Connected with MyD88 and TLR dependent induction of type I IFNs (IFN-α/β) DNA stimation(IKKβ)	Deteriorates obesity, hepatic steatosis, and the related inflammation.	In PCOS patients, IRF7 causes low-grade chronic inflammation and IR through androgen-dependent TLR4/IRF-7/NF-κB signaling, and metformin targets this pathway to alleviate symptoms; IRF7 also modulates MCP-1 transcription in adipose tissue, resulting in macrophage aggregation and pro-inflammatory effects

#### 3.2.2 Secreted frizzled-related protein 4 in IR

Secreted frizzled-related protein 4 (SFRP4) is a peptide hormone belonging to the SFRPs family, expressed in various tissues such as adipocytes, pancreas, and uterus (153–155). Elevated SFRP4 levels are observed in metabolic disorders linked to IR, like T2DM and gestational diabetes mellitus (GDM) (156, 157). SFRP4 plays a role in adipocyte differentiation, increasing adipokines, inducing oxidative stress in pancreatic cells with low antioxidant enzymes. Moreover, SFRP4 inhibits insulin extracellular secretion by reducing Ca2+ channel opening in pancreatic islet cells (153, 158). Wnt signaling (WNT5) showed a negative correlation with body weight in women with PCOS and controls (159). Acting as a Wnt signaling pathway antagonist, SFRP4 hinders Wnt proteins from binding to Wnt ligands or Fzd, thereby blocking the Wnt signaling pathway (160). These findings suggest that SFRP4 is directly or indirectly involved in the pathophysiologic pathway of IR.

#### 3.2.3 Afamin in IR

Afamin was biochemically characterized as a vitamin E-binding protein found in follicular fluid, suggesting involvement in the reproductive system, particularly in follicle maturation (161, 162).

Vitamin E, a crucial antioxidant that combats oxidative stress *in vivo*, suggests that Afamin likely participates in anti-apoptotic processes related to oxidative stress (163). Two studies reported elevated levels of hyperglycemia-induced reactive oxygen species (ROS) and activated NF-kB in PCOS patients, resulting in TNF transcription, with the most significant impact observed in obese PCOS patients (164, 165).

## 4 Immunotherapy

Since the immune mechanism of PCOS is rarely studied and the research of immunotherapy for PCOS is still in the preliminary stage, so in this part, we summarized the existing immunotherapy methods and looked forward to more research and exploration in this field.

### 4.1 Immunosuppressive therapy

Tacrolimus (FK506) is an 822 kDa lipophilic macrolide antibiotic known for its potent immunosuppressive properties. It has been shown to inhibit the production of IFN-γ and IL-2 by activated T cells, as well as the aberrant expression of chemokine CXC ligand. This inhibition effectively suppresses the inflammatory response in target organs, such as the ovary and adipose tissue in murine models, thereby promoting ovarian ovulation and providing protection against IR (166–168).

Isotretinoin, a derivative of vitamin A, acts as an effective antiproliferative and antikeratinizing agent with certain immunosuppressive effects. A prospective clinical study conducted in Egypt investigated the impact of oral isotretinoin administration in patients with PCOS. The study reported a significant reduction in both free testosterone levels and acne scores following treatment. However, it also noted a considerable increase in serum triglyceride and cholesterol levels among the patients (169). Importantly, the study did not include measurements of insulin and glucose levels. These findings suggest that while isotretinoin may effectively modulate hormonal levels in PCOS patients, it does not appear to alleviate symptoms associated with metabolic disorders.

### 4.2 Stem cell therapy

Mesenchymal stem cells (MSCs) significantly modulate the therapeutic immune response in a murine model of PCOS. Numerous studies have shown that MSC treatment markedly reduces serum expression of ovarian-specific genes and peripheral levels of the pro-inflammatory cytokine TNF-α in mice with PCOS. This effect may be mediated by the anti-inflammatory cytokine IL-10 (170–172). Peripheral blood flow cytometry analysis by Lamei Cheng et al. revealed that MSC treatment restored the proportions of macrophages and neutrophils in both peripheral blood and spleen to normal levels in the PCOS mouse model. Additionally, the ratio of pro-inflammatory M1 to anti-inflammatory M2 was appropriately adjusted. Variations in donor origin significantly affect the immunomodulatory effects of MSCs. Specifically, adipose-derived MSCs from obese or IR individuals show a markedly reduced ability to downregulate inflammatory factor expression and suppress CD4+ T cell activity. This reduced efficacy may result from intrinsic abnormalities in MSC function related to insulin resistance (173, 174). Currently, stem cell therapy targeting PCOS patients with IR, along with the underlying mechanisms affecting the immune system and stem cell abnormalities, has not progressed to the clinical research phase.

### 4.3 Links between treatment modalities and immunity

The clinical treatment strategy for IR induced by PCOS is comparable to that for type 2 diabetes. Lifestyle management is the preferred approach. Engaging in appropriate exercise and following a low glycemic index (LGI) diet can reduce waist circumference, total testosterone, low-density lipoprotein (LDL), fasting insulin, LDL cholesterol, triglycerides, and total cholesterol (175). The LGI diet decreases levels of growth hormone-releasing peptide while increasing glucagon levels. Physical activity and vigorous aerobic exercise enhance insulin sensitivity and androgen levels in patients with PCOS. Excessive fatty acid consumption and stabilization of hormone levels can effectively alleviate immune dysregulation in patients with PCOS. Lifestyle management is now widely promoted globally. Medications used as adjunctive treatments can effectively manage long-term complications.

Metformin (Met) is currently the primary therapeutic agent used for insulin sensitization in individuals with PCOS, while glucagon-like peptide-1 receptor agonists (GLP-1 RAs) are commonly employed in the treatment of T2DM. A study by Liao et al. investigated the efficacy of combining GLP-1 RAs and Met in reducing IL-6 and other molecules related to the inflammatory immune response, as evaluated through plasma proteomics (176). Additionally, Met has been shown to affect the levels of 11 cytokines in patients with PCOS, and its therapeutic efficacy is closely linked to androgen levels in these individuals (177).

The selection of pharmacological agents is crucial for improving insulin sensitivity and managing other metabolic disorders in patients with PCOS. As shown in the previous section, a significant association exists between the immune system and IR in patients with PCOS. Therefore, selecting medications that modulate inflammatory cytokine activity or inhibit the recruitment and proliferation of pro-inflammatory immune cells may offer a promising therapeutic strategy for future research.

In addition to medications, the incorporation of supplements may theoretically enhance the hypoglycemic effects of Met (e.g., sage, curcumin, quercetin, inositol, and various herbs) (178). However, aside from potential synergistic or additive effects with Met, no other known herb-drug interactions have been documented (179). Future therapeutic studies should investigate potential interactions between herbs or nutrients and drugs, as well as any immediate or long-term risks associated with these interactions.

## 5 Immunomarker

The immune system is a crucial system present at birth that continuously undergoes adaptive changes and regulatory functions throughout an organism’s life. Several comorbidities of PCOS are associated with low-grade inflammation, indicating altered immune function (180). Significant alterations in cytokine levels, including TNF-α, IL-6, IL-17, IL-18, and ultrasensitive C-reactive protein (CRP), have been observed in the peripheral blood of PCOS patients. These changes may result from an increased proportion of abnormal immune cells, such as pro-inflammatory M1 macrophages, CD4+ T cells, NK cells, and neutrophils (181–183). This aberrant manifestation is present in specific tissues of PCOS patients, including adipose tissue, the endometrium, and certain ovarian cells (180). Consequently, inflammatory molecules have been defined as biomarkers of PCOS. Among the PCOS phenotypes discussed in this article, levels of CRP, TNF-α, and IL-6 correlate with IR, body weight, and adiposity in PCOS patients (184). TNF-α is found in the subcutaneous adipose tissue of PCOS patients, where it accumulates with macrophages, differentiates, and increases in density along the CLSS (185). Conversely, IL-6 functions as a signal transducer and transcriptional activator in the inflammatory response to IR, regulating various target genes, including IRS, AP-1, and NF-κB, to promote systemic and local inflammatory responses. However, large clinical cohort studies correlating multiple inflammatory cytokines with IR incidence and severity in PCOS patients are still needed to statistically correlate cytokines with IR incidence and severity before further interpretation of the potential of cytokines as immune markers for IR can be made.

Abnormal proportions of immune cells may serve as biological markers for the detection of PCOS. Currently, the proportions of T-cells, CD4+ T-cells, and NK-cells are recognized as independent risk factors for the development of PCOS (181). Although the mechanism of B-cells remains incompletely understood, studies indicate that the number of B-cells inclined to differentiate into plasma cells is elevated in the peripheral blood of PCOS patients (186). This differentiation tendency is characterized by the presence of CD19+. Furthermore, administering an anti-CD19 antibody in PCOS mouse models has been shown to reduce serum TNF-α levels and macrophage infiltration in adipose tissue (187). This finding not only suggests the potential for B-cell intervention in PCOS treatment but also indicates that flow cytometry analysis of peripheral blood cells may serve as an early immunodiagnostic marker for the disease. Immunodiagnosis during the pre-morbid phase of PCOS lacks substantial research support. However, surface leukocytes, the most prevalent type of immune cells, have been examined in this population during this timeframe. Investigating the clustering of differentiation antigens among immune cells during this period, as well as analyzing cytokine levels in various tissues of patients with different subclinical phenotypes, could yield more credible clinical evidence for early immune markers in PCOS patients.

## 6 Outlook

In recent decades, significant progress has been made in identifying the causative factors of PCOS and clarifying its clinical symptom clusters; however, substantial gaps remain in our understanding of its biological mechanisms. Although IR is just one of the many clinical symptoms of PCOS, its long-term effects on the body are complex, including an increased risk of T2DM and cardiovascular disease. Various clinical interventions have been employed to manage PCOS and its related complications; however, without understanding the underlying causes of the disease, early intervention and delaying the onset of complications are not feasible. The immune system plays a crucial role in the pathogenesis of PCOS, characterized by its widespread distribution, diverse functions, and complex regulatory mechanisms. Investigating the detailed mechanisms of immune system actions is essential for tracing the origins and developing treatments for PCOS.

The prevalence of obesity in PCOS patients is 50-80% (188, 189). Mendelian randomization studies indicate a causal link between high BMI and PCOS risk, suggesting severe PCOS may increase BMI (190). Ethnicity, geography, and diet significantly affect the proportion of PCOS patients with high BMI globally. While the immunologic connection between high BMI, IR, and PCOS is well-studied, more research is needed on IR mechanisms in individuals with normal BMI.

Current research on the immune system in patients with PCOS primarily examines the distribution and abundance of immune cells and molecules. This includes the recruitment of pro-inflammatory M1 macrophages in peripheral blood and follicular fluid, alterations in the T1/T2 cell ratio, and an increase in B cells. Subsequently, the specific surface antigens of these immune cells are identified, and appropriate antibody therapies are administered. This approach aims to manage the progression of the inflammatory state, alleviate patient symptoms, and address the challenges associated with irregular and incomplete medication regimens. In addition to pharmacological treatment, standardizing lifestyle habits in PCOS patients can help reduce the risk and progression rate of the disease, particularly in those who are obese. Healthy dietary practices, regular exercise, and adequate sleep are effective strategies for reducing inflammatory factors in patients and modulating the distribution of immune cells. In recent years, there has been a surge in clinical trials investigating the effects of plant-derived products on PCOS. Natural compounds can antagonize certain pro-inflammatory cytokines and participate in the post-receptor signaling pathways of immune factors. They also enhance tissue cell sensitivity to insulin by influencing related molecules, including GLUT4 and IRS. Therefore, future investigations should focus on natural components to evaluate the purification effects of active ingredients responsible for the beneficial effects of these plants. Additionally, combining these with existing drugs could lead to the development of more effective formulations for hormone regulation and insulin enhancement in PCOS.

GMAS has identified over 20 genetic loci associated with PCOS; however, correlating these loci with the existing pathological mechanisms of PCOS remains a significant challenge. PCOS, an umbrella term for a group of clinical syndromes, shares common disease targets with T2DM, nonalcoholic fatty liver disease, and obesity (191). This overlap suggests potential mechanisms involving compensatory hyperinsulinemia, insulin resistance, systemic inflammation, cardiovascular disease, and pregnancy complications in PCOS patients. This suggests underlying mechanisms related to compensatory hyperinsulinemia, insulin resistance, T2DM, systemic inflammation, cardiovascular disease, and pregnancy complications in PCOS patients. Therefore, immunological studies of PCOS should extend beyond the female reproductive system to encompass metabolic changes throughout the body. This broader approach may uncover complex cellular interactions that facilitate the identification of biomarkers for predictive and targeted therapies, as well as improve predictions of therapeutic responses.

## 7 Conclusion

**Figure 4:**
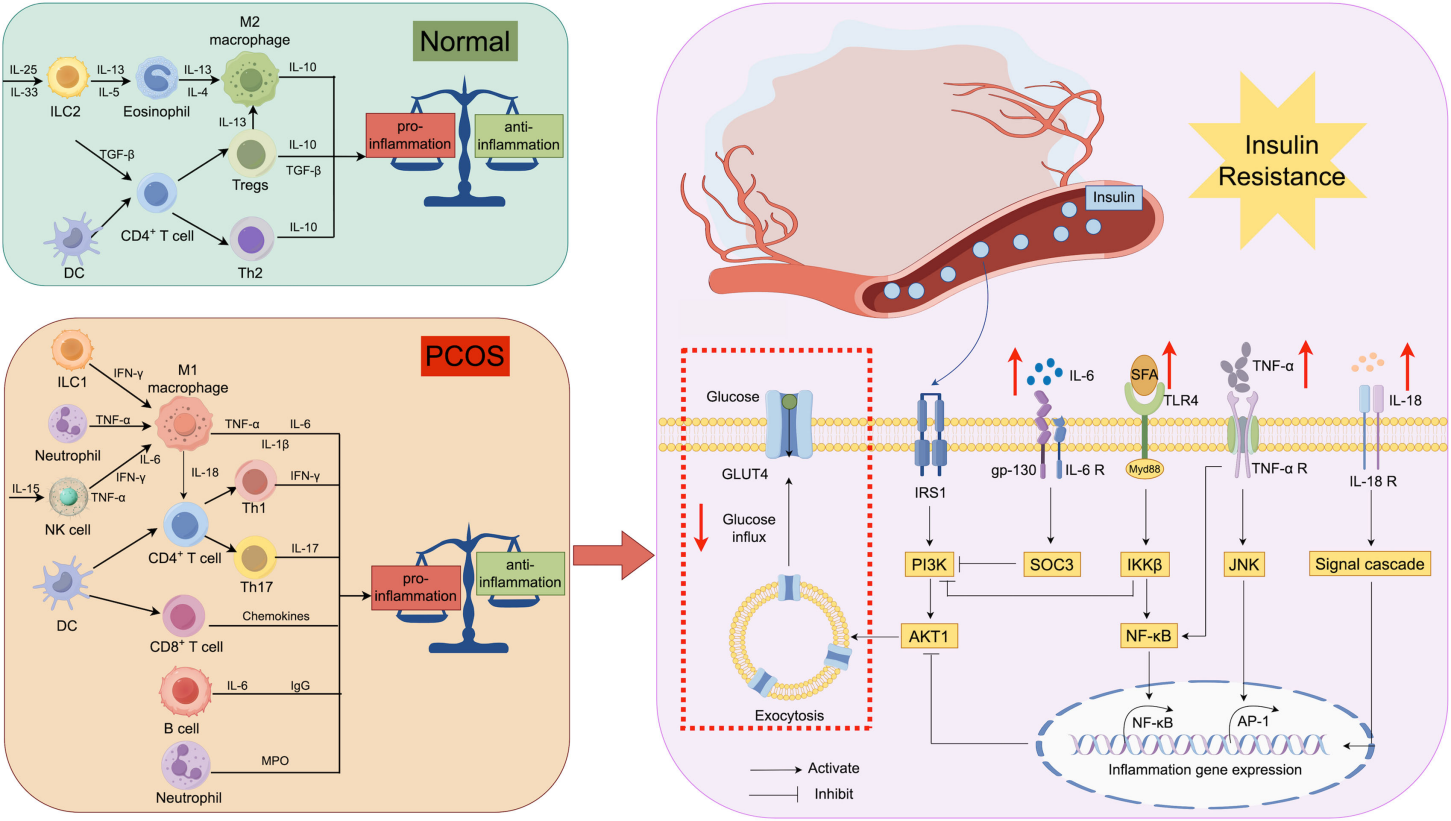
Relationship between immunity and insulin resistance: Immune cells secrete different cytokines in different states, and IL-6 affects the function of IRS-1 by regulating the expression of SOCS-3 in insulin receptor-containing cells. Meanwhile, TNF-α regulates the transcription of inflammatory genes through JNK and NF-kB signaling pathways. Excessive SFA in the body affects the nuclear translocation of NF-kB and inhibits IRS-1-related functions via IKKB.IL-18 has been shown to have an effect on the development of insulin resistance, but the exact pathway of action is unknown. All of these factors contribute to the blockade of insulin action and glucose glucose transport, resulting in IR. IL, interleukin; IRS, insulin receptor substrate; SOCS, suppressor of cytokine signaling; GLUT, glucose transporter protein.

PCOS, as a group of metabolic disorder syndromes with diverse and heterogeneous symptoms, has received widespread attention due to its sudden increase in prevalence in the general population. Imbalances in the immune system could potentially contribute significantly to the diverse symptoms of PCOS. This paper examined IR in PCOS, along with its related cellular and molecular mechanisms (**Figure 4**). Immune cells interact with immune molecules, triggering diverse signaling pathways in the body including NF-κB, JNK, and PI3K/AKT. This interaction influences cellular sensitivity to insulin and glucose transport efficiency by enhancing the transcription of inflammatory genes. Thus, assessing the expression of immune molecules and the ratio of immune cells in PCOS patients is crucial for evaluating their inflammatory status and preventing potential long-term complications symptomatically. Nonetheless, numerous immune-related molecules in PCOS patients have unidentified functions in insulin resistance, exhibiting intricate and diverse roles in pathways, as well as diverse functions of immune cells. Additional research is required to clarify the molecular biological mechanisms of the immune system in PCOS-related symptoms and to gradually integrate these findings into clinical practice. This integration aims to enhance the long-term prognosis and outcomes for patients, offering novel therapeutic approaches.
